# Improvement of an additively manufactured subperiosteal implant structure design by finite elements based topological optimization

**DOI:** 10.1038/s41598-021-94980-1

**Published:** 2021-07-28

**Authors:** Alberto Carnicero, Andrés Peláez, Andrés Restoy-Lozano, Isaías Jacquott, Ricardo Perera

**Affiliations:** 1 Institute for Research in Technology, ETSI-ICAI, Comillas Pontifical University of Madrid, C/ Alberto Aguilera 25, 28015 Madrid, Spain; 2 Department of Mechanical Engineering, Technical University of Madrid, Madrid, Spain; 3 Department of Oral and Maxillofacial Surgery, Principe de Asturias University Hospital, University of Alcala, Madrid, Spain

**Keywords:** Biomedical engineering, Mechanical engineering

## Abstract

To design a new subperiosteal implant structure for patients suffering from severe Maxillary Atrophy that lowers manufacturing cost, shortens surgical time and reduces patient trauma with regard to current implant structures. A 2-phase finite-element-based topology optimization process was employed with implants made from biocompatible materials via additive manufacturing. Five bite loading cases related to standard chewing, critical chewing force, and worst conditions of fastening were considered along with each specific result to establish the areas that needed to be subjected to fatigue strength optimization. The 2-phase topological optimization tested in this study performed better than the reference implant geometry in terms of both the structural integrity of the implant under tensile-compressive and fatigue strength conditions and the material constraints related to implant manufacturing conditions. It returns a nearly 28% lower volumetric geometry and avoids the need to use two upper fastening screws that are required with complex surgical procedures. The combination of topological optimization methods with the flexibility afforded by additively manufactured biocompatible materials, provides promising results in terms of cost reduction, minimizing the surgical trauma and implant installation impact on edentulous patients.

## Introduction

In the western world, the number of total edentulous patients is growing every year as a result of longer life expectancy^1^. Edentulism causes the resorption of the maxillary alveolar bone that produces functional and aesthetic alterations, decreasing the quality of life and making dental rehabilitation difficult^2^. Dental rehabilitation by means of conventional dental implants is impossible in chronic edentulous patients with severe resorption of the maxilla (Maxillary Atrophy Severe—AMS). They have traditionally been treated with bone graft surgery to reconstruct the alveolar process. This complex technique has a number of problems including unpredictable success rates in the medium and long term^3,4^ associated morbidity, length of treatment (owing to waiting times for the consolidation of the grafts and osseointegration of the implants, among other reasons), and the total cost. As a result current surgical practice with AMS patients favours the use of special implants, which are inserted in the malar bone. This technique is easier and faster, as it avoids the need for grafts and thus considerably reduces the treatment time. However, a high incidence of complications, mainly derived from the proximity or involvement of the maxillary sinus, has been reported^5^.

Recently, new design and manufacturing techniques have redeveloped the way edentulous patients are treated. Several Additive Manufacturing techniques, such as Electron Beam Melting^6,7^ (EBM), Selective Laser Melting^8,9^ (SLM), or Selective Laser Sintering^10–12^ (SLS), use biocompatible implantable materials to facilitate the manufacturing of implants with the required mechanical properties^13,14^. Individuals who may have lost .all their teeth and suffer from Cawood and Howell class V-VI bone atrophy are able to benefit from the progress made in the fields of Finite Element (FE) analysis and metal additive manufacturing. Additively Manufactured Sub-periosteal Jaw Implants or purposed Additively Manufactured Subperiosteal Implant Structures (AMSISs) associated to the surgical procedure and postoperative recovery^15^ may prove to be promising enhancement channels for severe atrophic cases stemming from maxillary bone loss and/or poor bone quality.

AMSISs consist of six abutments fixed to a main basal frame by six arms. The basal frame is fixed by the connection of four vestibular wings screwed to the midfacial pillars, the piriform and zygomatic buttress, respectively (where the bone thickness is most likely to ensure stability) and two lower arms also screwed to the palate. This methodology presents advantages in comparison with other implant alternatives^16^. However, different approaches can be used to improve the design of AMSISs, including the use of the FE method based on tools and additive manufacturing techniques.

The production of implant structures through additive manufacturing yields a large flexibility in the design of the implant since many shapes and geometries are more easily achievable than by using traditional or alternative production methods. Combining this with a finite element analysis, the critical points of the implant structure can be identified and reinforced while reducing the number of fastening points and optimizing their location. Moreover, the simplification of manufacturing restrictions facilitates the reduction of the contact surface between the implant and the maxillary bone, condensing the amount of bone subjected to contact and lessening the potential difficulties derived from bone loss. Eventually, this treatment may lead to shorter surgical incisions, reduce pain and surgery difficulties, facilitate screw fixation by osteosynthesis of the implant, and reduce post-operation recovery, enabling patients to adapt more quickly to their daily activities.

The research presented here derives from the collaboration between the Technical University of Madrid (UPM) and the University of Alcala de Henares (UAH). Taking as reference a real patient case and the AMSIS implant used to treat the malady in a first stage, the main goals were set to examine the specific original geometry of the applied implant and research possible geometrical improvement areas. The primary targets were a reduction of fastening screws, defined as eight on each side in the first model, and a reduction of the bone contact surface, which was being extensively used to communicate most of the different abutments and arms of the AMSIS. Ultimately, this design enhancement process will result in the easing of the discomfort suffered by the patient and the application of similar implant design techniques to this and future patient cases.

## Materials and methods

### Geometry and material

**Figure 1:**
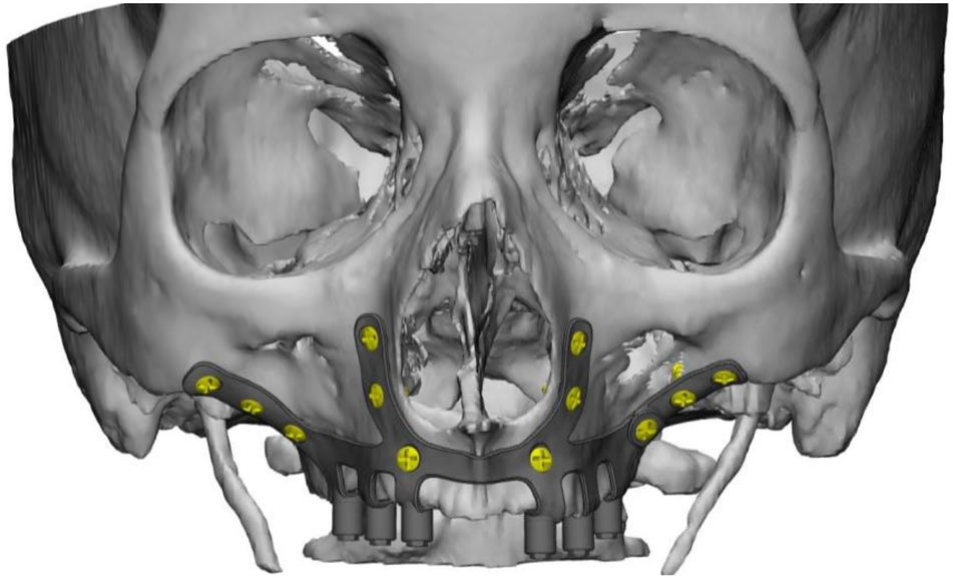
Base geometry attached to facial bones of the patient.

**Figure 2:**
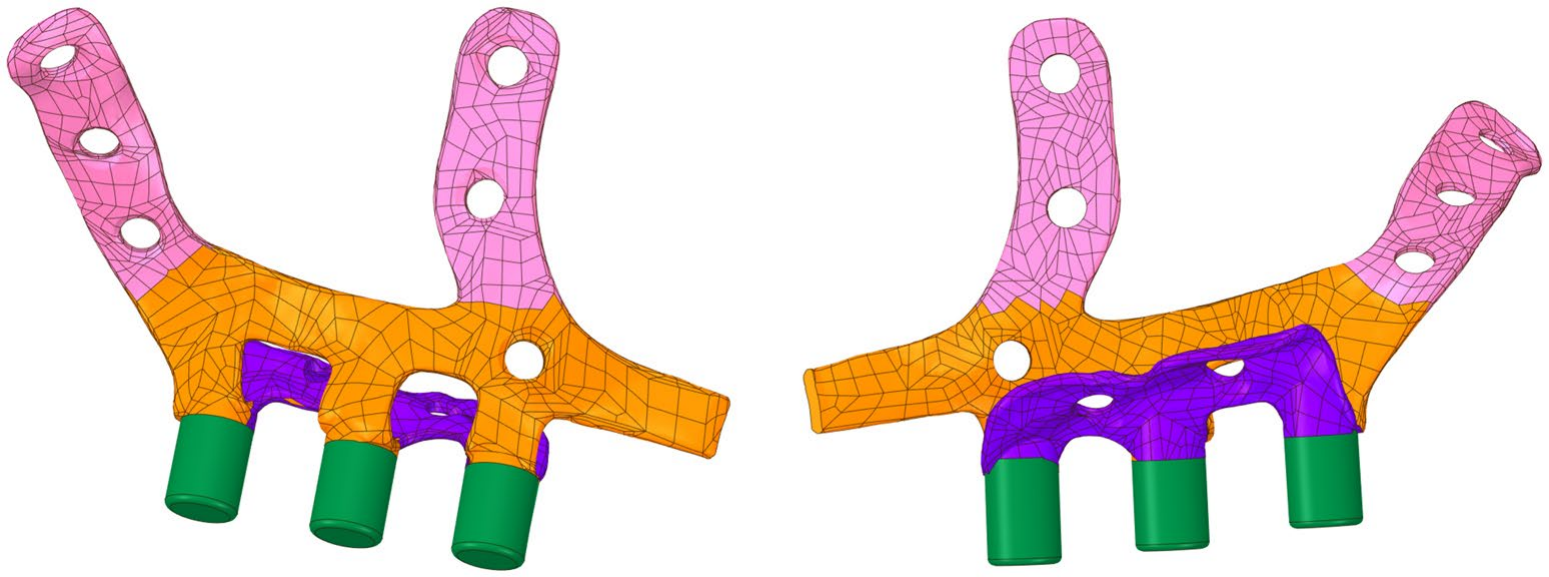
Base geometry, including frontal wings (pink), inferior palatal arms (purple), arms and posts (green). Figures edited with Inkscape 1.1, https://inkscape.org.

The base geometry (Fig. 1) consisted of two symmetrical annexes with six fastening points on each, divided into two frontal wings with three fastening points each, to be attached to the front zygomatic and maxillary bone. Furthermore, two lower arms with two fastening points each on the palate ensured the correct securing of the implant to patient’s maxilla. Each symmetrical annex of the original implant also contained three arms and posts, in green in Fig. 2, where the final denture was attached. With the aim of reusing the same design in terms of denture attachment in future applications, the position and connecting geometries of these posts in the implant were kept unchanged throughout the study. Consequently, the applicable portion of the geometry used in the optimization process did not include these arms and their corresponding posts.

The material used throughout the experiment was a Ti6Al4V titanium alloy, which was chosen owing to its biocompatibility with surrounding bones and soft tissues. The ultimate strength value of the material was determined as 920 MPa and the Young’s modulus and Poisson ratio were set to 116 GPa and 0.31, respectively^17^. The fatigue strength was selected as 200 MPa based on the additively manufactured nature of the implant as researched by Greitemeier et al^18^. According to this study, no fatigue damage would happen until 10^7^ cycles if the stress values are kept below the mentioned limit. The implant was fixed with tapping screws of 5 or 6 mm in length and 2.1 mm in diameter in a previously drilled 1.5 mm hole. The screws were not modelled in this research.

### Model

The implant was modelled from CT (computed tomography) images scanned from the original implant. The CT scans were converted to STL format using the software 3DSlicer 4.11 (https://www.slicer.org/). This format was read afterwards using the 3D CAD modeling software Ansys SpaceClaim 2020R1 (www.ansys.com). A three-dimensional reconstruction of the images was produced with a surface triangularization technique. For this purpose, a redefinition of edges and surfaces from scanned images was conducted in order to build a simplified model covering most of the volume of the original geometry. Throughout the process, special care was taken to keep the surfaces of the implant in contact with the bone as faithful as possible to the original model to avoid possible future incompatibilities with the patient’s remaining maxillary bone. This same procedure was performed to treat the resulting geometry after each optimization stage. Finally, before this model could be read into a FE program for mesh generation and subsequent strain and stress analyses, the geometry was divided into 2 sub-geometries according to an oral sagittal plane, and a symmetry plane was established to simplify the calculations.

FE simulations and optimizations were made within the Ansys Workbench 2020R1 framework (www.ansys.com). FE analysis provided a comprehensive examination of the areas of the geometry subjected to higher and lower stress levels according to the different bite force scenarios defined. Based on these results, the target portions of the implant to be modified were identified using engineering and medical criteria, and the geometry was adapted to a new lower volume—optimized implant model.

In terms of meshing, tetrahedral elements were used because of their better adaptability to the numerous surfaces and edges derived from the model generation. Furthermore, the options *patch independent* and *quadratic element order* were defined to enhance the mesh generation and fidelity of the meshed model by ignoring certain less important points and improve the mesh surface curvatures. Different refinement and defeaturing options were tried but did not prove to be beneficial. Consequently, the mesh was generated using Solid186 element which supports irregular meshes as well as possible orientations of different nodes or elements. A body sizing refinement of average 1 mm and a face sizing refinement of average 0.5 mm were introduced for each of the optimization models and most areas of the implant except the three posts used for denture attachment. This configuration resulted in an average of 100,000 nodes and 60,000 elements composing the different models subjected to optimization and a mesh quality parameter between 0.8 and 0.85 in most of the meshed models. This quality index measures if the shape of the tetrahedral is close to a regular tetrahedron or not. A value of 1 indicates a perfect regular tetrahedron while a value of 0 indicates that the element has a zero or negative volume. Therefore, the obtained values can be considered suitable.

While further mesh refinements resulted in mesh failure considering the geometrical difficulties mentioned above, the consistency and reproducibility of the results associated with the used mesh controls reinforced the choices made in terms of meshing.

The study also investigated the position and allocation of loads that would reflect modelling and meshing decisions. Since the implant is attached to the denture through the posts, it is reasonable to think that the load should be transmitted to the implant structure via these posts. Since an optimization of the posts was not sought, each of the complete post cylinders was used for the load application. Furthermore, the fixation of the implant demonstrated that the holes created for the fastening screws were the best position to impose movement restrictions, as different alternatives were tried involving the use of friction between the implant and the bone as well as different fixation conditions on those holes.

### Boundary conditions and loads

There is no specific consensus regarding the suitable boundary conditions to be used when simulating the stress distribution suffered by maxillary and zygomatic implants during chewing actions, either in terms of implant movement restrictions or the magnitude and direction of the applied forces.

Regarding the movement restriction of the implant, Mommaerts^15^ assumes a displacement distribution restriction simulating the constraint introduced by the contact between the implant and the bone. In other investigations (Ishak et al.^19^, Saini et al.^20^, Demenko et al.^21^, Field et al.^22^ and Geramy et al.^23^), the maxillary and jaw bones are also simulated, and some material properties are assumed for their cancellous and cortical parts, often noting different values of these properties. However, only Mommaerts^15,16^ research is devoted to a similar type of implant to the one used in this study, as the rest of the references are focused on other types of implants where the different bones and their composition and layers play a more critical role. In this research, movement constraints were applied on the fastening holes existing in the different wings through the nodes located on the attachment hole circumferences. To test this approach, the displacement of the structure was monitored during the application of the different loading cases. Taking the displacement values of similar mentioned studies as a reference, lower values than those were obtained in most of the simulated cases, validating the constrained model.

With regard to the definition of load cases, Mommaerts^15^, Demenko et al.^21^, Kaman et al.^24^ and Liu et al.^25^ assumed a total maximum chewing force of around 150 N in the vertical direction for their implant and tooth bite studies. Other researchers [Ishak et al.^19^, Saini et al.^20^, Miyamoto et al.^26^, Ujigawa et al.^27^, Cattaneo et al.^28^] included a 300 N vertical force value derived from the chewing support of the masseter muscle. Ishak et al.^19^, Miyamoto et al.^26^ and Ujigawa et al.^27^ also considered the action of a horizontal force of 50 N, in order to simulate the impact of an imperfect distribution of the chewing load among the three spatial directions. Some studies have tried unsuccessfully to offer a defined criterion that may explain the divergences in the measurements and simulations explained. By contrast, Koc et al.^29^ attempt to elucidate the influential factors of the bit force measuring method used by Ferrario et al.^30^ and Shinogaya et al.^31^. Tuxen et al.^32^ and Shinogaya et al.^31^ detail the evaluation deviations that are related to sex or age.

Two load cases were used in this study to cover partially the divergences commented in the previous paragraph. These two approaches try to reflect the chewing dynamics of patients recovering from implant installation surgery and the chewing needs of a standard meal (where a maximum force is not applied in every single chew). Firstly, a standard chewing loading case composed of a vertical force of 150 N and a horizontal force of 50 N (Table 1) was compared to the Fatigue Strength limit of the material to estimate the implant life over a reasonable average daily usage. Secondly, a critical chewing loading case composed of a vertical force of 450 N and a horizontal force of 50 N was contrasted with the strength of the structure under a reduced number of loading cycles to provide an overview of the implant response under unfavorable conditions. In both loading cases, the resultant forces were applied to the three posts, simulating load transfer from the denture and considering that the load is uniformly distributed through the internal metal structure of the implant.

**Table 1:** Loading cases by type of force and displacement constrains.

Case	Force (N)		Displacement (constrained/unconstrained)							
	Vertical	Horizontal	1	2	3	4	5	6	7	8
1	150	50	C	C	C	C	C	C	C	C
2	450	50	C	C	C	C	C	C	C	C
3	150	50	C	C	U	C	C	U	C	C
4	150	50	C	C	C	U	U	U	C	C
5	150	50	U	U	U	C	C	C	C	C

**Figure 3:**
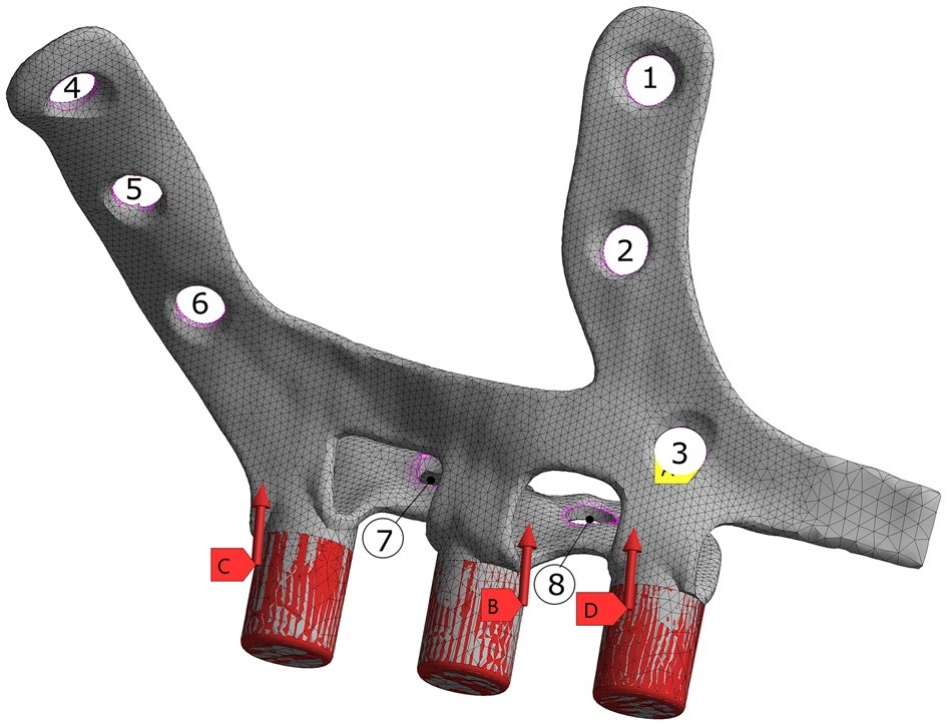
Loads and anchorage lines numbers included in Table 1.

Additionally, to analyze the effect of screw loosening due for instance to bone weakness, three different constraints were tested under standard chewing loads (case 1). Table 1 shows the five cases with their different boundary conditions. Figure 3 shows the load location and the anchorage line numbers (from 1 to 8).

### Optimization

The optimization process was performed in two stages and therefore involved three models: the initial reference model, a first model derived from the reference model resulting from the first optimization stage, and a second model following the same process as the first one. The first phase of the optimization focused on refining the coarser impractical areas, while the second one concentrated on more specific surfaces to adjust the final model for manufacturing purposes searching for smoother edges and transitions between surfaces. In both stages, the five load cases shown in Table 1 were considered and their results helped to define the resulting geometry for each of the further steps. Solid Isotropic Material with Penalization method (SIMP) has been used as the topology optimization method^33^. This well-known method penalizes the stiffness of the elements with a lower contribution to the objective function. This function minimizes the weight of the structure without exceeding the fatigue stress limit of the material. As there are no convergence issues, the parameters of the method have not been changed from the default values (maximum number of iterations 500 and a convergence accuracy 0.1%).

As commented previously, the implant posts where the denture is attached and the holes for the fastening screws were not subjected to the optimization process (green zone in Fig. 2). In this sense, the target areas for volume reduction were described following criteria related to the study goals, i.e., the comfort of the patient via the reduction of the contact surface between the implant and the maxillary bone and the localization and reinforcement of the structural weak points.

The main objective of the procedure was to minimize the weight of the implant while keeping a Von Mises stress constraint lower than 200 MPa for loading case 1 and between 250 and 400 MPa (depending on the maximum von Mises stress of the previous simulation) for cases 2–5 (Table 1).

Finally, a manufacturing constraint was added to the optimization calculations for production purposes. Due to the additive process restrictions, all areas of the structure had to have at least 1 mm of thickness to ensure that no issues would appear during the implant fabrication.

## Results

A first evaluation of the base geometry of the titanium structure placed and screwed on the patient maxilla (Fig. 1) revealed a large improvement margin in areas surrounding the fastening holes, the surfaces connecting the two external wings, and those linking the middle denture connecting post with the external wings. The results for the standard bite loading case gave a maximum stress concentration near the lower fastening point of the external left wing and a maximum equivalent stress value 32% lower than the fatigue limit of the material. The critical loading and fastening cases gave results which did not compromise the structural integrity of the implant.

**Figure 4:**
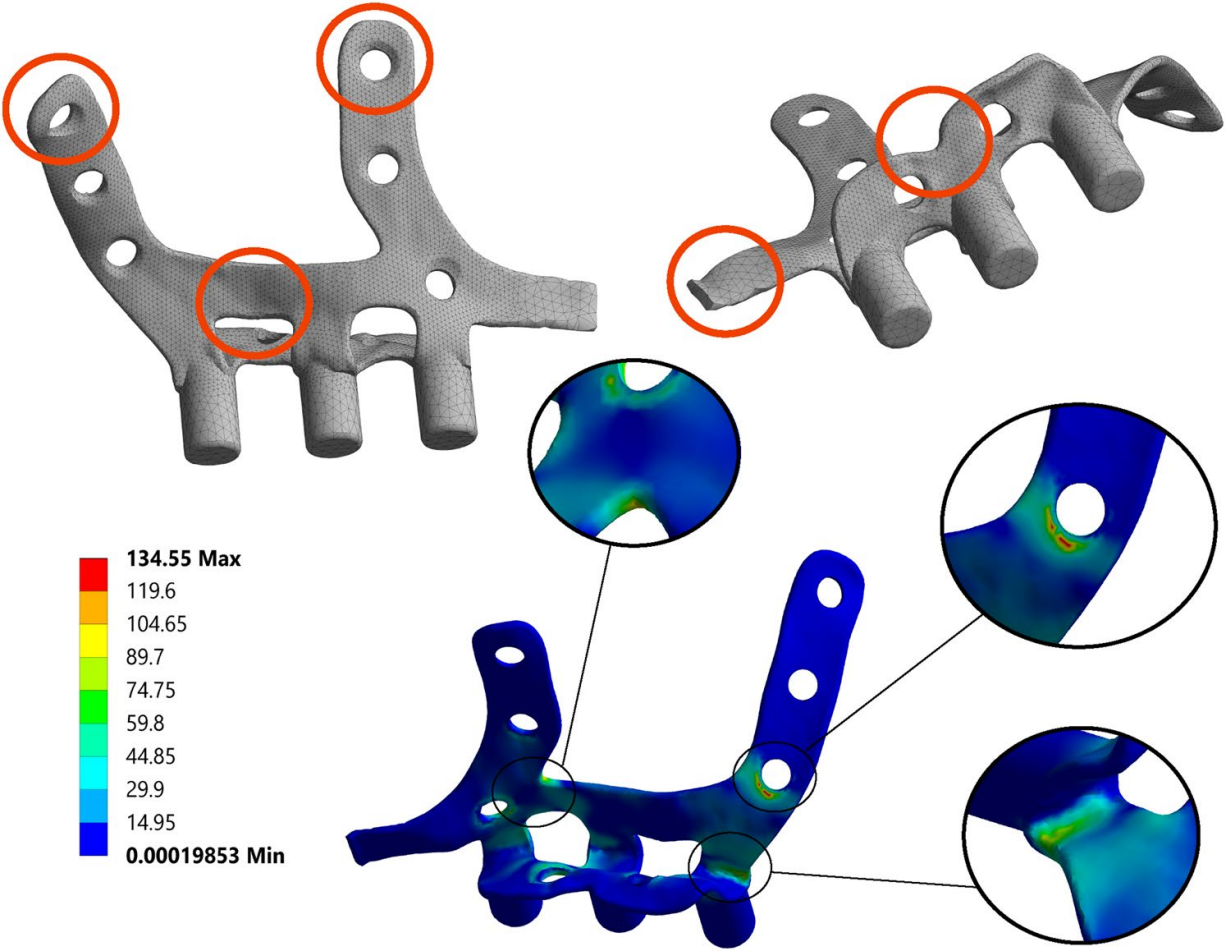
Overview of frontal and palatal base geometry (top left and top right) including optimization areas considered on the first stage (in orange) and, Von Mises stress [MPa] distribution for load case 1 (bottom center). Maximum stress 134.5 MPa.

Based on these results, a first geometrical optimization was performed (Fig. 4). The upper fastening holes of the external wings, the surfaces connecting the two external wings and the two palatal wings, and the arm used for linking the two sides of the geometry divided by the symmetry plane were reduced in size (areas marked in orange in Fig. 4). The immediate effects were that the implant insertion method was modified to two independent solids and the connection of the upper fastening screws was avoided. The maximum stress point was located at the same area in most cases and models independently of the boundary conditions, which was clearly a symptom of the strong and weak points of the geometry.

**Figure 5:**
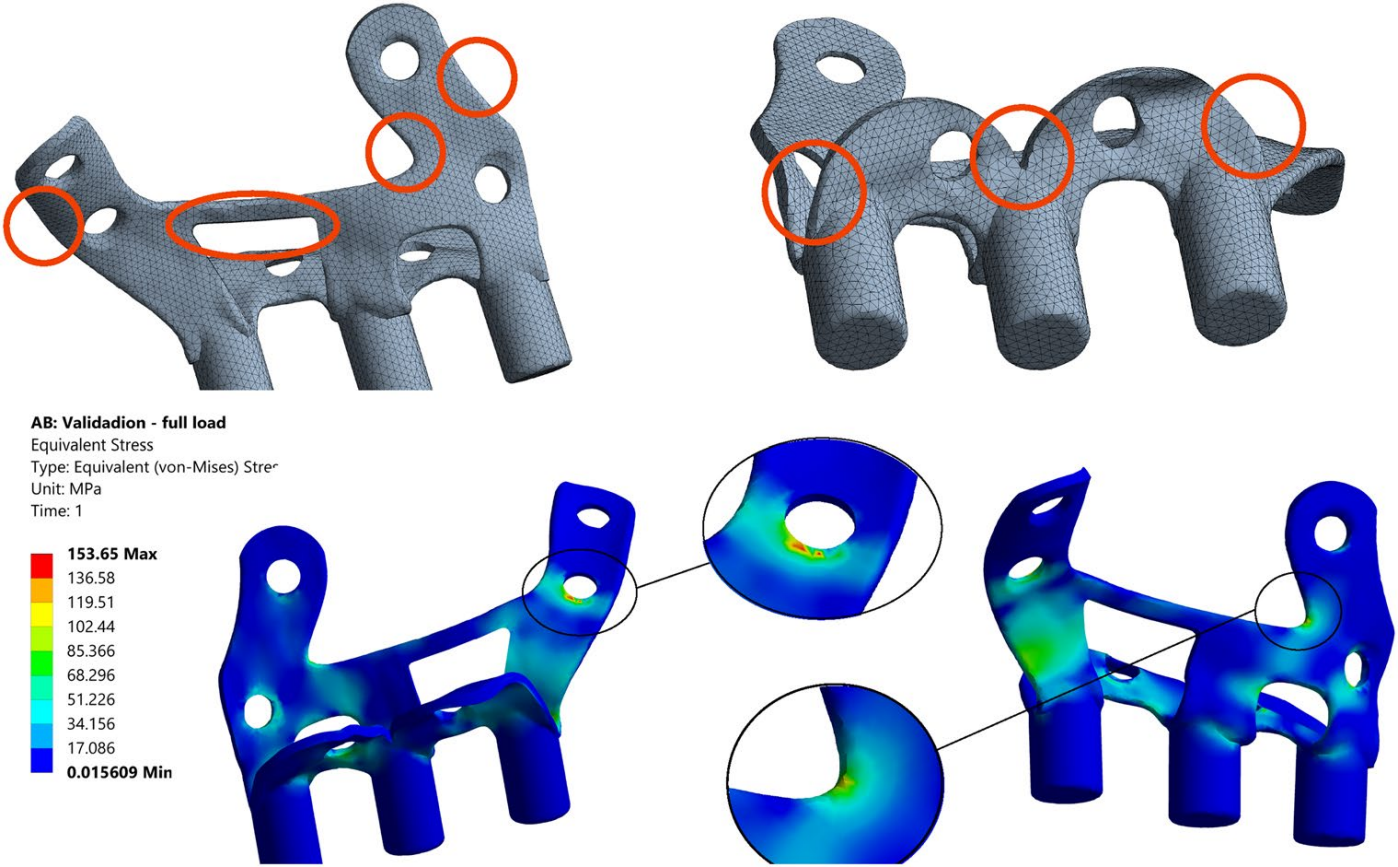
Overview of Opt1 geometry (top left and top right) including the optimization areas considered in the second stage (in orange). Stress distribution of load case 1 (bottom center), indicating the location of the maximum Von Mises stress (153.6 MPa).

A new model, referred to as Opt1, was built after the first optimization stage (Fig. 5). This model provided an 18.56% reduction in volume with respect to the initial model. When subjected to the five loading cases of Table 1, Opt1 showed an expected increase of the maximum equivalent stress values, but to a level at which the fidelity of the implant was not in danger.

Subsequently, starting from Opt1, a new optimization stage was carried out providing a new optimized model, Opt2. The execution of a new optimization phase based on the stress results of Opt1 revealed new zones in the surface of the external and internal wings that could be subjected to reduction (marked in orange in Fig. 5) and identified some new stress concentration points on some edges or surfaces, once the structure had been weakened from the previous step.

**Figure 6:**
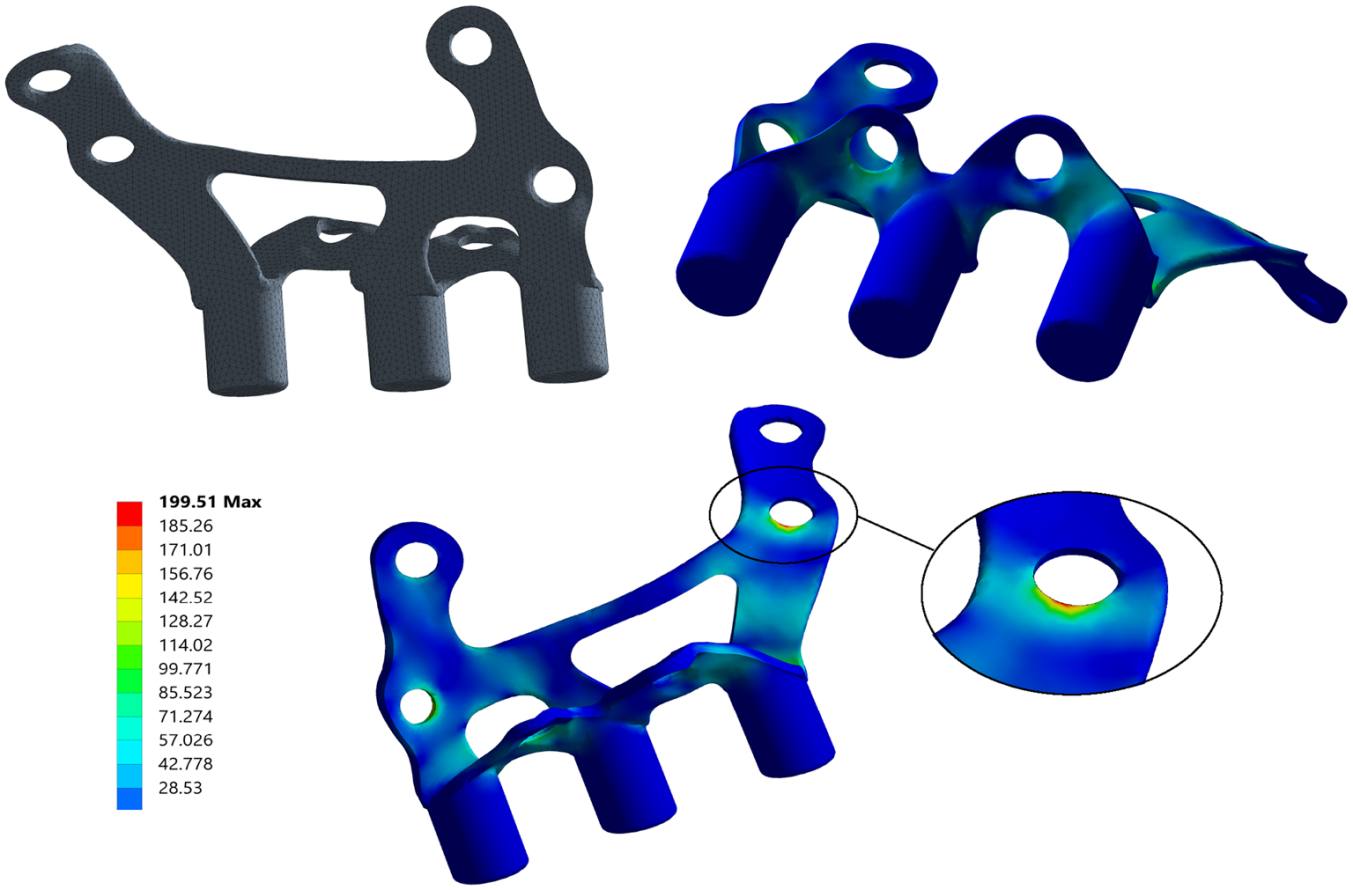
Overview of final Opt2 geometry (top left) and Von Mises stress [MPa] distribution of Case 1 (bottom center and top right). Maximum stress 199.5 MPa.

The newly created model, Opt2, exhibited a volume reduction of 11.21% with respect to Opt1, and a total reduction of 27.68% with regard to the base geometry. This model (Fig. 6) also included a smoothing of the edges and surfaces, which can enhance the design and reduce possible stress concentrations. As per the standard bite loading (case 1), Opt2 exhibited a maximum equivalent stress of 199.51 N, just under the established limit of 200 N for fatigue life, which ensures the resistance of the implant to at least 10^7^ chewing cycles following material tests.

For the final optimized model, the critical bite load simulation yielded a stress level equivalent to around 10^5^ chewing cycles when compared to the S–N curve of the material. According to the estimations provided by Farooq et al.^34^ for bite cycles per meal, this means that more than 151.5 meals might be completed under critical bite loading conditions, which is an acceptable value given the surgery and recovery processes of the patient. The maximum stress obtained in the critical fastening loss cases (cases 3–5) is equivalent to 2 × 10^6^, 6 × 10^6^ and 5 × 10^5^ chewing cycles, that is, 3030, 9090 and 757 meals, respectively. Assuming an average of 3 long meals per day, a period of 36 weeks seems more than reasonable to fix the fastening loss. Accordingly, the stress results are judged to be satisfactory and Opt2 is considered optimal as a result of this study.

As a summary, Table 2 shows the maximum Von Mises stresses for each of the loading scenarios and models.

**Table 2:** Maximum Von Mises stress [MPa] for each of the loading cases and models.

Geometry	Case 1 (MPa)	Case 2 (MPa)	Case 3 (MPa)	Case 4 (MPa)	Case 5 (MPa)
Base	135.55	280.97	213.34	148.71	142.29
Opt1	153.65	326.96	203.48	235.26	196.44
Opt2	199.51	395.03	275.78	237.56	317.87

Finally, the effect of the topological optimization on the anchoring of the implant was computed to check if some fastening had experienced an unacceptable overstress. As shown in Table 3, only slight modifications (between − 11.7% and + 8.9%) in the reaction forces appeared between the initial and the optimized model. Figure 6 shows the location of the different anchorage points in agreement with Table 3. Therefore, the effect of the geometrical modification did not significantly increase the mechanical effort on the maxilla and malar bones.

**Table 3:** Comparison of reaction force [N] at the eight anchorage lines of Fig. 3. Reactions in the optimized model have no significant differences with the base case.

Load case	Model/point	1	2	3	4	5	6	7	8
1	Base	2.1	29.33	58.83	0.13	3.32	49.21	28.21	36.18
	Opt2	–	28.1	58.82	–	3.6	49.88	28.78	34
	Diff [%]	**–**	**−** 4.1	0	**–**	6	1.3	2	**−** 5.9
3	Base	6.6	**−** 0.42	Free	10.35	33	Free	51.87	49.26
	Opt2	–	32.3	Free	–	34	Free	46.55	43.51
	Diff [%]	**–**	35.7	**–**	**–**	3.6	**–**	**−** 10.26	**−** 11.7
4	Base	2.9	1.39	64.18	0.12	3.3	52.05	17.39	Free
	Opt2	–	31	62.82	–	2.8	51.58	16.23	Free
	Diff [%]	**–**	31.7	**−** 2.12	**–**	**−** 16	**−** 0.9	**−** 6.67	**–**
5	Base	2.1	2.29	59.71	0.08	3.8	54.22	Free	26.01
	Opt2	–	**−** 0.42	59.23	–	4	51.91	Free	28.34
	Diff [%]	**–**	32.3	**−** 0.8	**–**	6.4	**−** 4.26	**–**	8.96

## Discussion and conclusions

3D finite element analysis is a numerical stress analysis technique that is widely used to study engineering and biomechanical problems. Its combination with new manufacturing techniques, such as Additive Manufacturing, results in considerable improvements in the design and performance of implants when compared with more traditional approaches. In this study, an optimization of the original design of a sub-periosteal jaw implant has been carried out using these tools. The optimized implant structure has an individualized titanium network shape, with six implant-like connections fixed directly to the bone of the upper jaw by means of micro-screws. This design makes it possible to produce a biocompatible structure with optimal biomechanical characteristics for the subsequent fixation of a fully functional dental prosthesis.

The topologic optimization leads to a new the design of AMSIS that improves performance, reducing the mass of titanium material used (25% less) and therefore the cost, the number of screws (four fewer than in the original one), and the surgical time required for its fixation, as well as the trauma to both the soft tissues and the bone of the recipient patient. All this facilitates surgery and allows a postoperative period with less pain and inflammation, maintaining the functionality of the structure, which clearly benefits the patient.

The rehabilitation of atrophic maxillae by means of this type of structure without the need for bone regeneration or reconstruction has important advantages:

- Elimination of donor area morbidity as bone grafting is not required.
- Possibility of ambulatory realization.
- Reduction of the time required for prosthetic loading.
- Possibility of using immediate provisional prosthesis
- Possibility of immediate loading
- Decrease in the number of face-to-face patient consultations.
- Possibility to treat medically compromised patients
- Possibility to treat patients with sinus pathology
- Treatment of irradiated patients
- Possibility of treatment of large maxillary defects (maxillectomies).

The study proves that the use of these computational tools increases the comfort and quality of life of edentulous patients that may eventually benefit from sub-periosteal jaw screwed implant structures.

It is important to point out that the design guidelines presented in this paper are general. Further refining processes according to each individual particularity may result in further optimization processes to accommodate the resulting geometry to the likeness of the final receiver without reducing fidelity.

Nonetheless, considering the previously mentioned benefits of this design technique and the potential unforeseen difficulties of the use of the implant, the improvement of the location and type of fastening attachments through the geometrical optimization of the implant may bring some solutions to those potential issues.
